# Variation, during Shelf Life, of Functional Properties of Biscuits Enriched with Fibers Extracted from Artichoke (*Cynara scolymus* L.)

**DOI:** 10.3390/nu15153329

**Published:** 2023-07-26

**Authors:** Francisco José San José, Montserrat Collado-Fernández, Pino P. Álvarez-Castellanos

**Affiliations:** 1 Centro de Innovación y Tecnología Alimentaria de La Rioja, CTIC-CITA La Rioja C/Los Huertos 2, 26500 Calahorra, La Rioja, Spain; 2 Departamento de Biotecnología y Ciencia de Los Alimentos, Universidad de Burgos, Plaza Misael Bañuelos, sn., 09001 Burgos, Spain; montcol@gmail.com; 3 Departamento de Ingeniería Agrícola, Universidad Católica de Ávila, UCAV, C/Canteros, sn., 05005 Ávila, Spain; mperez.alvarez@ucavila.es

**Keywords:** polyphenols, antioxidant activity, shelf life, pea fiber, fiber-rich powders of artichoke (FRPA), functional biscuit

## Abstract

To boost revaluation of industrial by-products of artichoke, this research tries to determine the stability throughout storage of phenolic compounds and their antioxidant activity in biscuits enriched with fiber-rich powders extracted from b y-products of artichokes (FRPA). To determine the most stable extraction method, biscuits were formulated with FRPA extracted by two different environmentally friendly extraction solvents: water (W) and a solution of 1% CaCl_2_∙5H_2_O (CA) and compared with biscuits made with pea fiber (P) and control biscuits (B) without fiber added. Initially and during storage, the biscuits enriched with FRPA (W, CA) showed a higher content of bioavailable polyphenols and antioxidant activity compared to the control biscuits (B) and the reference fiber (P, pea fiber). In conclusion, FRPA are an excellent source of bioavailable fiber with antioxidant activity, but especially the FRPA extracted with 1% CaCl_2_∙5H_2_O (CA), and they could present a good alternative to the use of pea fiber.

## 1. Introduction

In recent years biscuits with high fiber content have been established as one of the most stable sectors in the functional food industry. The population of the western world attempts to consume products that supply fiber, which their lifestyle and current diet does not provide [1,2]. Therefore, the enrichment of food products like biscuits, with new sources of dietary fibers obtained from vegetal by-products, could be a good alternative to complement the needs of the fiber of the population through the consumption of a daily product [3]. Moreover, it could yield the environmental management of these by-products profitable and reduce illnesses, suffered by populations in industrialized countries, such as gastric cancer, diabetes, constipation, obesity, irritable bowel syndrome, etc. [4,5,6,7,8,9,10].

Since vegetal by-products have a rich content in antioxidant compounds, the fiber-rich powders obtained from vegetal by-products could be a good alternative to enrich food products with antioxidant compounds. In their turn, these could both reduce processes of rancidification or fat oxidation during storage of the biscuits [11] and complement the diet with these nutrients. In the literature, several studies can be found related to the production and extraction of high antioxidant activity fiber-rich powders from different vegetal by-products [12,13] such as carrot peels [14], outer leaves of cabbage [15], cacao [16], grape seeds and pomace [17,18], sesame husk and rice brand [19], asparagus [20], cereals [21], and finally, and more related to our study, from artichoke [22,23,24].

Regarding the application of compounds rich in fiber with a high antioxidant power, in oven-baked products like biscuits [25], in recent years products have been used from extracts of fruit and wild plants [26] and of tea leaves [27], and finally, and more related to our study from, artichoke [28,29].

In regard to the present study on the production of biscuits with antioxidant fiber-rich powders, we can highlight the studies by Vitali et al. [30] who utilized fiber from apple by-products and Ajila et al. [31] who employed mango by-products.

Currently, several vegetal fibers are already marketed for the enrichment of biscuits with high fiber content, as is the case of beetroot (Fibrex^®^), chicory (Raftilosa^®^), and pea [32]. However, we are not aware of studies regarding the formulation of biscuits with fiber-rich powders obtained from the revaluation of artichoke by-products, generated by the canning industries. There are some studies on the functional properties and content of fiber-rich powders of artichoke (FRPA) extracted by different methods [33], which support the possible use of these fiber-rich powders in the formulation of novel products.

The objective of this study was to determine the phenolic compounds and the antioxidant activity of biscuits produced with fiber-rich powder of artichoke, extracted from the by-products of artichoke, and to investigate their evolution during storage. Biscuits were made with FRPA, extracted by two distinct methods: water (W) and 1% CaCl_2_∙5H_2_O (CA), and compared with biscuits prepared with pea fiber. For the control biscuits, no fiber was added. Two types of storage were carried out: one at room temperature (25 °C, seven months) and another accelerated (45 °C, six months).

To test whether these antioxidant compounds could maintain their effect and bioactivity throughout the storage period and the digestive process after their consumption, they were subjected to a model of human digestion and an extractive chemical process.

## 2. Materials and Methods

### 2.1. Sample Preparation

A standard formulation like the fiber-rich biscuits sold in the Spanish market was used [34], with slight modifications in the percentages of the ingredients. Three different batches were prepared for each type of biscuits on various days. For FRAP, samples of artichoke by-products (*Cynara scolymus* L.) variety (Blanca de Tudela) were from the canning company GVTARRA S.A. (Villa Franca, Navarra, Spain). The vegetable by-product, mainly external bracts, and peduncle obtained by the blanching process and core extraction, were sent to our laboratory within 2–4 h after its production. Once centrifuged and removing the remains of blanching water, the by-product rested stored frozen (−14 °C) in polypropylene bags by lots of the same weight (500 g) until extraction and chemical analysis. FRPA were obtained in our laboratory by liquid extraction with distilled water (W) and with a solution of 1% (*w*/*w*) CaCl_2_∙5H_2_O (pH 6.5) (CA) following the method applied by Fuentes-Alventosa, [35] with some modifications. We applied two different extraction solvents: distilled water (W) and a solution of 1% (*w*/*w*) CaCl_2_∙5H_2_O (pH 6.5) in distilled water (CA) and using a proportion (1:1) solute: solvent (1 L of solvent per 1 Kg of frozen by-products of artichoke (−10 °C)). Dividing the load into four batches of 5 min each at maximum speed and renewing the solvent proportion each time, Each solvent extraction method was performed in triplicate using a 2 L Blender (Hallde, Kista, Sweden).

After extraction, the solute was strained and wrapped in absorbent paper, and the solvent was removed by compression. 

Finally, the different solutes were dried in a convection oven (Zanussi, Madrid, Spain) at 65 °C for 6 h or until reaching a constant weight. Then, aliquots of 50 g of dry by-products were ground using a coffee grinder MC6250, 110W (Solac, Vitoria, Spain) at maximum speed for 5 min.

Thus, the biscuit types formulated were as follows: control biscuits without fiber (B), biscuits with pea fiber (P), the commercial fiber purchased from the local trader of the brand Garuda International named PEAFIBFG^®^, and biscuits enriched with FRPA obtained in our laboratory by liquid extraction with distilled water (W) and with a solution of 1% (*w*/*w*) CaCl_2_∙5H_2_O (pH 6.5) (CA) Each batch of biscuits was analyzed separately and in triplicate. Table 1 shows the formulation used for the preparation of the biscuits. The biscuits were formulated with 4% of fiber (fiber weight/wet weight). For the control biscuits (B) the fiber was replaced by the same weight of whole meal flour.

The dough is kneaded with a pilot mixer for 10 min at minimum speed. Subsequently, the dough was laminated to a thickness of 5 mm, using a manual sheet pasta machine. The round form of the biscuits was obtained manually, using a cutting cylinder (die) of 60 mm in diameter. 

After making eight evaporation holes in the dough with a fork, the biscuits were baked in an electric convection oven at a temperature of 180 ± 10 °C for 17 min. Afterwards, the biscuits were cooled at room temperature (18–20 °C) and wrapped in heat-sealed bags of high-density polyethylene. Until analysis, the biscuits were divided into two groups as a function of the storage conditions. As a control for each group of biscuits, the values were taken at time 0, which was the day after the preparation.

### 2.2. Storage

The plastic-wrapped and coded biscuits were stored in two incubation chambers (Incudigit Selecta, Barcelona, Spain), with constant temperature and humidity. One of the chambers simulated storage at room temperature (25 °C, RH 55%), while the other simulated conditions of accelerated storage (45 °C, RH 55%) [36,37], forcing the appearance of defects in the biscuits by the increase in temperature. The biscuits stored under ambient conditions were analyzed monthly, during the seven months of the duration of this study, while the biscuits stored under accelerated conditions were analyzed every three months until completion of the six months of storage.

By comparing the results of both storage procedures, we intend to understand under which ambient conditions the defects are detected, generated under accelerated storage.

### 2.3. Determination of Moisture

Moisture (M) in the biscuits was determined by the method described by Nollet [38] with slight modifications. Biscuit samples (250 mg) were weighed in nonuplicate on an analytical balance (Mettler Toledo, Columbus, OH, USA, and they were dried for 24 h at 90 °C. Subsequently, moisture was calculated as the difference in weight.

$$(M=(\left(Wet\;weight-Dry\;weight\right)*100)/weight\;sample).$$

### 2.4. Determination of the Functional Properties of the Biscuits

**Figure 1:**
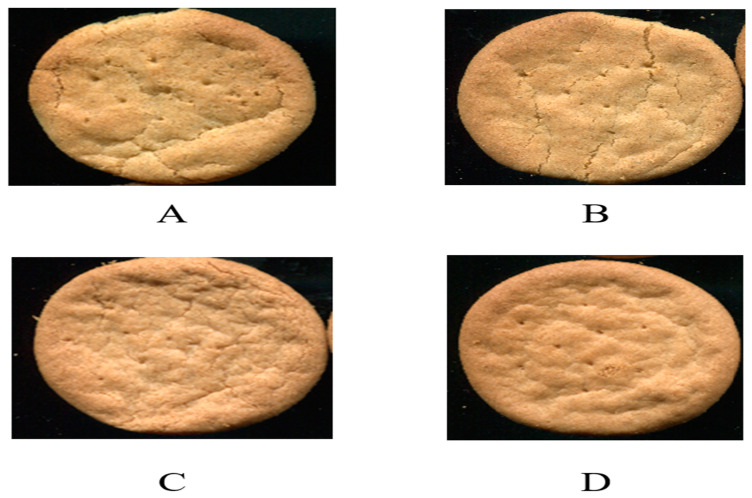
Scanned picture of the biscuits after oven-baking. (**A**) Biscuit W, (**B**) Biscuit CA, (**C**) Biscuit B, (**D**) Biscuit P. Biscuit codification (B (without fiber), P (pea fiber), W (water), CA (1% CaCl_2_∙5H_2_O).

**Figure 2:**
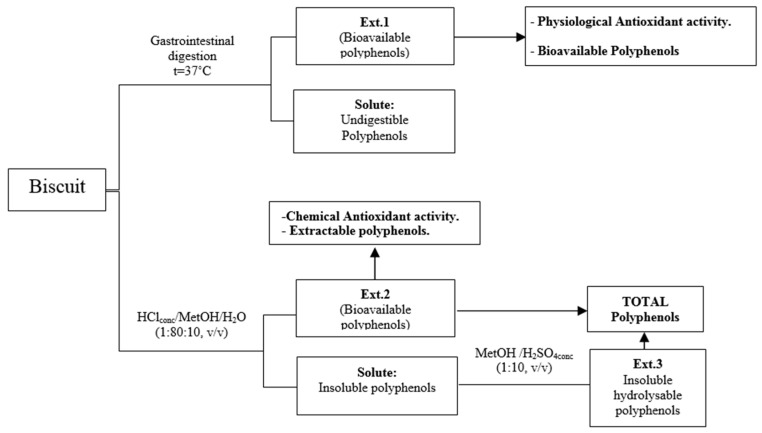
The Schematic procedure of analyses. Vitali et al. [30].

To investigate the effect of the functional properties of the biscuits enriched with FRPA (Figure 1), the working method proposed by Vitali et al. [30] was followed, with some modifications (Figure 2). A human digestive model was performed, with which an extract (Ext.1) was obtained, and the content of bioavailable polyphenols was determined. A chemical extraction of the polyphenolic compounds was performed with which two extracts were obtained for the determination of the polyphenolic content: extractable (Ext.2) and hydrolysable (Ext.3). All the extractions were carried out in triplicate.

#### 2.4.1. Model of Human Digestion

For the digestion of the biscuit samples, the method of Glahn et al. [39] was utilized. It was proposed for the study of the availability of iron in food products in an in vitro digestion/Caco-2 cell culture model, with small modifications to adapt it to biscuit samples. This model attempts to imitate the phases of the human digestion such as acid digestion in the stomach, neutralization with pepsin and pancreatin, and, finally, intestinal absorption. Once the digestion model was terminated, the digestion extract (Ext.1) was stored frozen at −4 °C until analysis (polyphenols and antioxidant activity).

Yun et al. [40] also used this method to predict the influence of phenolic compounds on iron bioavailability. Other authors have used this method to study bioavailability of functional compounds such as polyphenols, [41] or carotenoids [42].

#### 2.4.2. Extraction of Polyphenolic Compounds from a Food Matrix

The extraction method described by Vitali et al. [30] proposed for samples of food products with little moisture like cereals or biscuits was utilized. The extraction of soluble phenols from the biscuits was performed by an extraction with a mixture of HCl conc/methanol/water (1:80:10, *v*/*v*) during 24 h, at 20 °C, with constant shaking. After centrifugation (3500 rpm, 10 min) the supernatant (Ext.2) was frozen at −4 °C until analysis. The extraction conditions applied are not able to extract the total phenolic content of the food products, since the phenols are bound to structures of the fiber, which must hydrolyze. The extraction of the insoluble phenols was carried out by the procedure of Hartzfeldet al. [43]. Briefly, the pellet of the first extraction was subjected to a second extraction process with a mixture of methanol and sulfuric acid (10:1, *v*/*v*) during 20 h, at 85 °C. (Ext.3). After centrifugation (3500 rpm, 10 min) the supernatant was stored frozen at −4 °C, until analysis.

#### 2.4.3. Polyphenols

The content of total polyphenols was calculated as the sum of the fractions of extractable (Ext.2) and hydrolysable (Ext.3) polyphenols, while for the content of polyphenols with physiological antioxidant activity after digestion Ext.1 was analyzed. Polyphenols were estimated using the method proposed by Singleton and Rossi [44], measuring absorbance at a wavelength of 765 nm. The results were compared with a calibration curve, previously carried out with gallic acid, using concentrations between 0 and 0.1 mg of gallic acid/mL and were expressed as mg of gallic acid per mL of extract.

#### 2.4.4. Antioxidant Activity

For the determination of the total antioxidant activity of the biscuits, Ext.2 and Ext.3 were used, while for the antioxidant activity after digestion, Ext.1 was used. Several methods were performed for the analysis of antioxidant activity, ABTS.^+^, FRAP, and DPPH.

##### ABTS.^+^ Assay

This test was determined according to the method of Re et al. [45]. The variation in the absorbance of the samples was measured at a wavelength of 732 nm, using a spectrophotometer, after 7 and 30 min of incubation of the extracts in the dark.

The calibration curve was prepared using Trolox as a standard. Antioxidant activity of the extracts was expressed as TEAC, which is the antioxidant activity in Trolox equivalents (mEq Trolox/g dry weight of biscuit).

##### DPPH Assay

This test was determined according to the method of Brand-Williams et al. [46], with some modifications [36]. Absorbance in the extracts was measured at a wavelength of 517 nm using a spectrophotometer. The calibration curve was performed with concentrations between 25 and 800 mM of Trolox, obtained by different dilutions of a Trolox stock solution of 4 mM (1 mg/mL). The results were expressed as TEAC.

##### FRAP Assay

The FRAP test was carried out according to Iris Benzie and Strain [47] with some modifications [36]. Absorbance in the extracts was measured at a wavelength of 593 nm using a spectrophotometer. The results were expressed as TEAC.

### 2.5. Statistical Analysis

Statistical analysis was performed using the software Statgraphics Centurion XVI 16.1.03 version (Statpoint Technologies Inc., Warrenton, VA, USA). Results were expressed as mean Values ± standard deviations. The differences between the variables and samples were analyzed using one-way ANOVA and in the event of significant differences (*p* < 0.05) the post-hoc LSD test.

## 3. Results

### 3.1. Moisture

**Figure 3:**
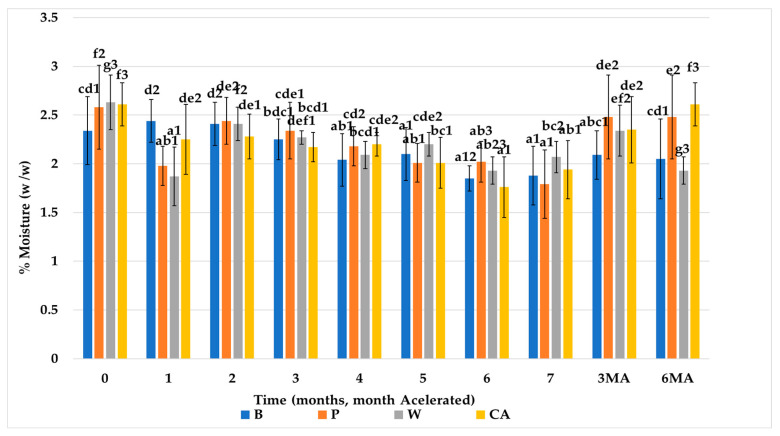
Moisture evolution. Values are mean ± standard deviation (SD). (*n* = 3); significant (*p* < 0.05) differences between storage time within biscuit types (columns) are indicated with different letters, significant (*p* < 0.05) differences between biscuit types (rows) are indicated with different numbers. Storage codification, letter (fiber source), number (months stored at room temperature), AM accelerated month). Biscuit codification (B (without fiber), P (pea fiber), W (water), CA (1% CaCl_2_∙5H_2_O).

Figure 3 shows the results obtained of moisture, for the different types of biscuits studied, throughout the storage period at ambient and accelerated temperature. The biscuits formulated with artichoke fiber (W, CA) had higher initial moisture content than the control biscuits (B) and the biscuits with pea fiber (P). During storage at room temperature, the moisture content of the biscuits decreased, with some fluctuations. During storage at accelerated temperature, all groups of biscuits with fiber showed a significant reduction in moisture content.

### 3.2. Phenolic Content and Bioavailability and Antioxidant Activity of the Biscuits Studied

**Figure 4:**
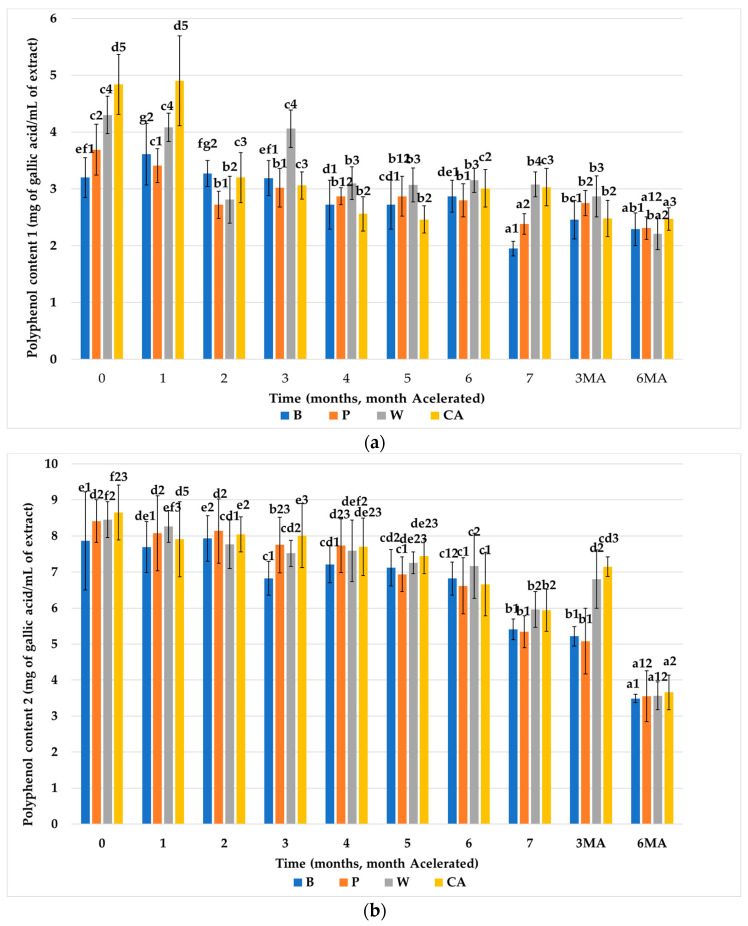
Polyphenol content (mg of gallic acid/mL of extract) of the biscuits studied (B, P, W, CA) during storage: (**a**) from Ext.1, (**b**) from Ext.2 + Ext.3. Values are mean ± standard dev. (*n* = 9); significant (*p* < 0.05) differences between storage time within biscuit types (columns) are indicated with different letters, significant (*p* < 0.05) differences between biscuit types (rows) are indicated with different numbers. Storage codification, letter (fiber source), number (months stored at room temperature), AM accelerated month). Biscuit codification (B (without fiber), P (pea fiber), W (water), CA (1% CaCl_2_∙5H_2_O).

To evaluate whether the fiber-enriched biscuits may be a good source of bioavailable polyphenolic compounds. The polyphenolic content was evaluated of the extracts obtained after having subjected the biscuits to a human digestion model (Ext.1) Figure 4a. In this way we also wanted to investigate whether the usual approach of estimating soluble phenols in foodstuffs accurately reflects the content of bioavailable polyphenols.

The total phenolic content was calculated as the sum of the extractable (Ext.2) and hydrolyzable (Ext.3) polyphenolic fractions, according to Pérez-Jamiréz and Saura-Calixto [48]. The results obtained from the content of polyphenols in the control biscuits without fiber (B) and in the biscuits prepared with the fiber of artichoke (W, CA) and pea fiber (P), throughout the storage period, are shown in Figure 4b. The initial polyphenolic concentration of biscuits subjected to a human digestion model was higher in all biscuits prepared with fiber than in the control biscuits without fiber (B), and the groups with fiber showed significant differences amongst each other. Therefore, the increase in polyphenol content is attributed to the enrichment of the formulation of the biscuits with vegetal fiber.

The total polyphenol contents (Ext.2 + Ext.3) of the biscuits studied are shown in Figure 4b. It can be observed that the content of total polyphenols of all biscuits showed a great difference from the one obtained after digestion of the biscuits (Ext.1). This is due to the liberation of the insoluble polyphenols, bound to the plant structures, in the process of acid extraction. Similar to what was found for Ext.1, the initial polyphenol content of the biscuits prepared with fiber was significantly higher than of the control biscuits without fiber.

Consequently, the increase in polyphenol content is the result of the polyphenolic content provided by the vegetal fiber added to the formulation. The initial contents of the biscuits formulated with FRPA (W, CA) and pea fiber (P) was significantly higher than the initial content of biscuits P. Throughout storage at room temperature, the concentration of extractable polyphenols (Ext.2 + Ext.3) significantly decreased in all groups of biscuits (≈30%) until the end of the storage period (seven months). The biscuits enriched with FRPA (W, CA) had a higher content of total polyphenols than the other biscuits (B and P), showing a significant difference at the end of the storage period. Similarly, a decrease in the polyphenolic content is observed for all types of biscuits during accelerated temperature storage. When comparing the storage at room and accelerated temperature, it is noticeable that the total polyphenol content decrease occurred after three months of accelerated storage. This decrease was similar to the reduction observed after seven months of storage at room temperature for the biscuit groups that did not contain FRPA (B and P).The consistency of our results was roughly estimated and confirmed by comparing them with data for polyphenol contents of samples with a similar source or technological process to ours [30,49,50,51,52] or the same food matrix (biscuit) [53,54,55], confirming too that baking does not impact the composition of polyphenols from FRPA and that baking the biscuits a suitable carrier for FRPA to preserve polyphenols.

The polyphenol content of the biscuits without fiber is mainly the result of the polyphenol content of the wheat bran [52,56]. It is noteworthy that at the start of storage, the biscuits formulated with artichoke fiber (CA and W) had a significantly higher polyphenol content than the biscuits formulated with the reference fiber (P).

At room temperature, the polyphenol content of Ext.1 of all groups of biscuits showed a significant decrease throughout the storage period. The greatest decrease was observed in biscuits B (39%) and the smallest decrease was found in biscuits W (28%). The biscuits enriched with FRPA maintained a significantly higher content of polyphenols than the biscuits enriched with pea fiber. However, at the end of accelerated storage, no significant differences in polyphenol content were observed between the control biscuits and the rest of the biscuits, except for the biscuits enriched with artichoke fiber CA, which had the highest content of total polyphenols. 

The results obtained suggest that the products enriched with FRPA could be considered food products rich in polyphenols and could contribute to complementing the requirements of daily intake (DRI) of dietary polyphenols. Curiously, although polyphenols are essential dietary components, at present their DRI’s have not been established yet [57,58].

### 3.3. Antioxidant Activity

The main ingredient of the biscuits is wholemeal flour, which contains phytochemicals deriving primarily from the layer of bran of the whole cereal grain. These compounds present antioxidant properties, and therefore biscuits could be considered for its own right as a foodstuff with potential antioxidant properties. To determine the antioxidant activity of the biscuits were used different analytical different methods. The radical-sequestering activity was elevated when the methods ABTS.^+^ and DPPH. were used, based on the discoloration of the cation radical. The FRAP method was used to determine the ferric reducing power of the analyzed samples.

To compare the concentration of antioxidants liberated from the food matrix during human digestion conditions. The antioxidant activity was determined in two different extracts (Ext.1) with the antioxidants that are solubilized by the solvent (MeOH: water: HCl) of the chemical extraction (Ext.2).

#### 3.3.1. ABTS.^+^

**Figure 5:**
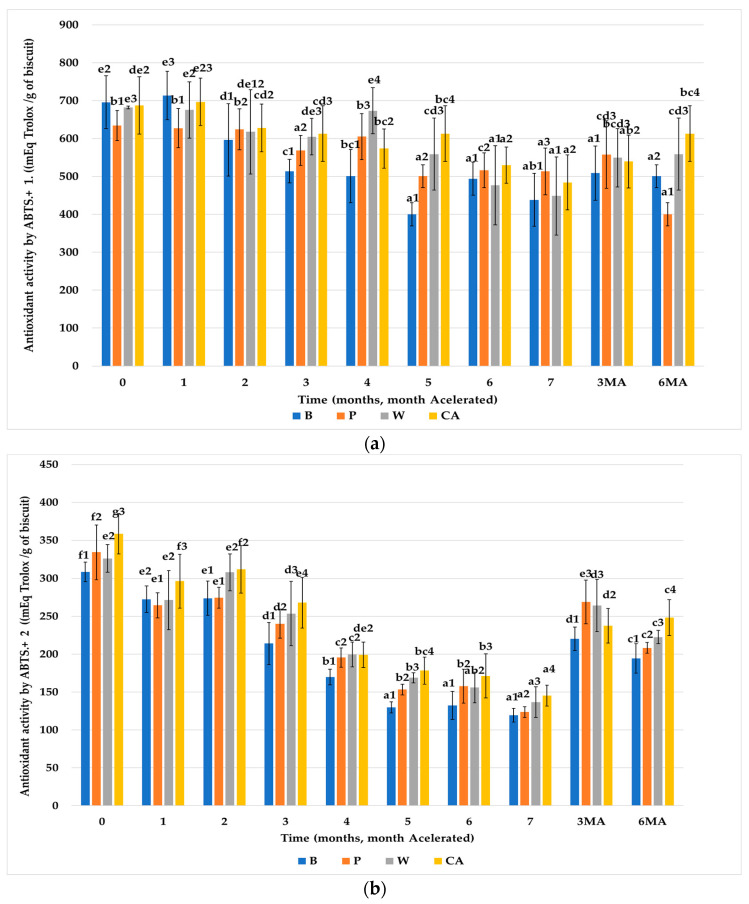
Evolution of antioxidant activity by ABTS.^+^ ((mEq Trolox/g of biscuit) of the biscuits studied (B, P, W, CA) during storage. (**a**) From Ext.1. (**b**) From Ext.2. Values are mean ± Standard Dev. (*n* = 9); significant (*p* < 0.05) differences between storage time within biscuit types (columns) are indicated with different letters, significant (*p* < 0.05) differences between biscuit types (rows) are indicated with different numbers. Biscuit codification (B (without fiber), P (pea fiber), W (water), CA (1% CaCl_2_∙5H_2_O). Storage codification, letter (fiber source), number (months stored at room temperature), AM accelerated month).

Figure 5a shows the results obtained for the antioxidant capacity in Ext.1 by the ABTS.^+^ method throughout the storage period at room temperature. As can be observed, the biscuits enriched with FRPA (W and CA) initially showed higher antioxidant activity than the other two types of biscuits. In particular, the control biscuits (B) had the lowest antioxidant activity. The control biscuits (B) had antioxidant activity because they contained natural antioxidant compounds found in sunflower oil (vitamin E) and the antioxidant content of the wheat bran fiber [25]. Additionally, the biscuits enriched with Pea fiber (P) and FRPA (W, CA) further increased their antioxidant activity due to the contribution of each fiber. 

These primary differences leveled off throughout the storage period for all groups of biscuits. However, the antioxidant activity of the biscuits formulated with (P, CA, W) was more stable throughout storage, especially for the results from Ext.2 (Figure 5b). 

The antioxidant activity of the extracts by chemical extraction (Ext.2) was higher in the biscuits prepared with fiber (P, W, CA) than in the biscuits without fiber (B), due to the contribution of the vegetal fibers to the content of the biscuits. Initially, a significantly higher antioxidant activity was observed of the FRPA than the reference fiber (P). At the end of storage at room temperature, the antioxidant activity values of the biscuits formulated with vegetal fibers (P, W, CA) leveled off, but the trend of significantly lower values in the control biscuits without fiber (B) remained. 

Comparing the results of the last months at room temperature with biscuits stored under accelerated conditions, we observed that an increase in antioxidant activity in both the physiological (Ext.1) and chemical (Ext.2) extracts. This effect may be due to the temperature of the accelerated storage, which would generate the same antioxidant compounds produced during baking. Reports in the literature suggest that the increase in antioxidant activity could be attributed to the formation of certain compounds from the Maillard reaction or to oxidation products of lipids under the influence of high temperature, which can occur during oven-baking [59,60,61,62]. During accelerated storage (45 °C), after three months an increase was observed in the antioxidant activity of the extracts by chemical extraction (Ext.2) of the biscuits, which could be due to the formation of Maillard compounds, as mentioned before. However, after six months of accelerated storage antioxidant activity of all the biscuits decreases, which is probably the result of oxidation of the fats. Nevertheless, the antioxidant activity of biscuits with fiber (P, W, CA) was still significantly higher than of the biscuits without fiber (B).

When comparing the results obtained for the extracts of the digestion model (Ext.1, Figure 5a) with those of the chemical extraction (Ext.2, Figure 5b). It is evident that the free radical antioxidant activity of the digestive extracts of the biscuits (Ext.1) was significantly higher than that of the chemical extracts (Ext.2). This difference could be caused by the contribution of hydrolysable antioxidant compounds liberated by the acid digestion step that imitates the action of gastric Chlorhydric acid (HCl) in combination with the digestive enzymes (pepsin and trypsin). Pepsin acts on proteins and converts them into peptones, while trypsin converts peptones into polypeptides, digesting the food matrix and enhancing the release of functional compounds.

#### 3.3.2. DPPH

**Figure 6:**
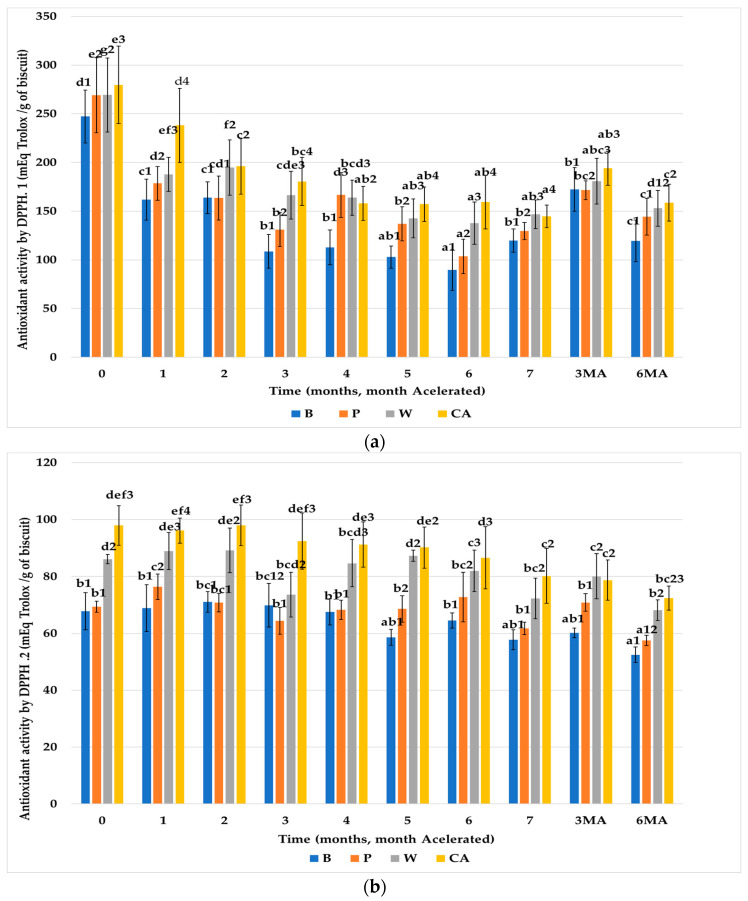
Evolution of antioxidant activity by DPPH. (mEq Trolox/g of biscuit) of the biscuits studied (B, P, W, CA) throughout storage. (**a**) From Ext.1. (**b**) From Ext.2., values are mean ± standard dev. (*n* = 9); significant (*p* < 0.05) differences between storage time within biscuit types (columns) are indicated with different letters, significant (*p* < 0.05) differences between biscuit types (rows) are indicated with different numbers. Biscuit codification (B (without fiber), P (pea fiber), W (water), CA (1% CaCl_2_∙5H_2_O). Storage codification, letter (fiber source), number (months stored at room temperature), AM accelerated month).

Figure 6a,b shows the results of antioxidant activity measured by DPPH. analysis of the control biscuits without fiber (B), with FRPA (W, CA), and with pea fiber (P), subjected to a model of human digestion (Ext.1). It is observed that the control biscuits (B) and the biscuits formulated with the reference fiber (P) were very similar to each other and significantly lower than the biscuits enriched with FRPA (W and CA). Thus, the chemical content of the FRPA contributed in a significant way to the antioxidant effect of the formulation of the biscuits compared to the reference fiber (P). Throughout storage at room temperature, the antioxidant activity of all the biscuits tended to decrease. However, the antioxidant activity of the biscuits formulated with FRPA (CA, W) was more stable. On the contrary, an increase in antioxidant activity is observed in the biscuits subjected to accelerated storage.

Regarding the results of the chemical extraction (Ext.2), the primary antioxidant activity of the biscuits prepared with FRPA (W, CA) was significantly higher than that of the control biscuits (B) and the biscuits formulated with reference fiber (P), due to the enrichment with vegetal fiber. Equal to what was observed with the ABTS.^+^ method, during storage at room temperature, the values of antioxidant activity of all the biscuits analyzed by DPPH. decreased with time of storage. The biscuits formulated with FRPA (W, CA) maintained higher antioxidant activities throughout the storage period. When comparing the results of antioxidant activity of the Ext.1 of the biscuits with the Ext.2, it is also confirmed that the process of digestion liberated hydrolysable antioxidants, reactive with DPPH. from the plant structures, and, therefore, Ext.1 showed higher values of antioxidant activity compared to Ext.2, for all biscuits. The trend throughout storage time was maintained at both storage temperatures. 

#### 3.3.3. FRAP

**Figure 7:**
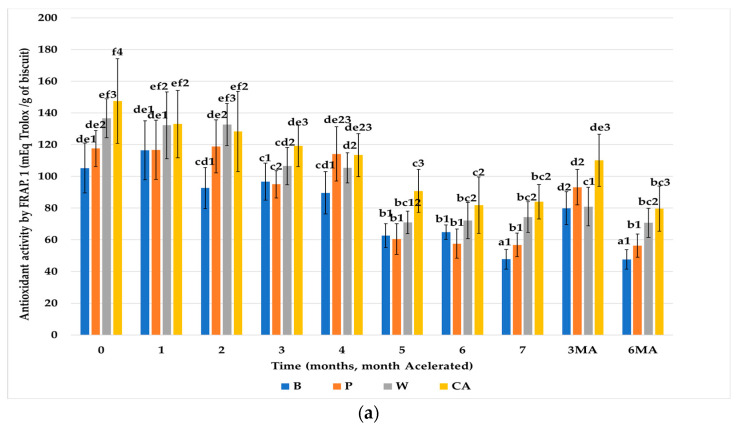

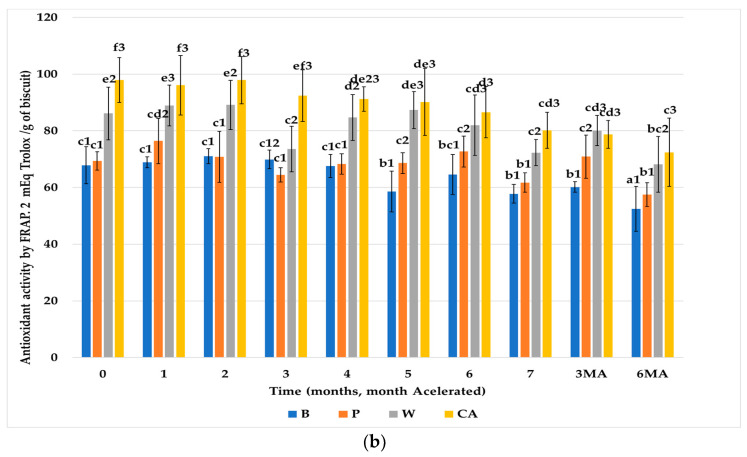
Evolution of antioxidant activity by FRAP (mEq Trolox/g of biscuit) of the biscuits studied (B, P, W, CA) during storage. (**a**) From Ext.1. (**b**) From Ext.2. Values are mean ± standard dev. (*n* = 9); significant (*p* < 0.05) differences between storage time within biscuit types (columns) are indicated with different letters, significant (*p* < 0.05) differences between biscuit types (rows) are indicated with different numbers. Biscuit codification (B (without fiber), P (pea fiber), W (water), CA (1% CaCl_2_∙5H_2_O). Storage codification, letter (fiber source), number (months stored at room temperature), AM accelerated month).

The results obtained from antioxidant activity, using the FRAP method, of the biscuits subjected to a model of human digestion (Ext.1) are shown in Figure 7a It can be observed that the control biscuits, without fiber (B), and the biscuits formulated with reference fiber (P) had similar initial values, which were significantly lower than those of the biscuits enriched with FRPA (W and CA). These differences between the different types of biscuits were maintained throughout storage at room temperature. These results show the same trends as those obtained with the other analysis techniques of antioxidant activity and once more indicate that this higher antioxidant activity was due to the antioxidant content of the FRPA (W and CA) added to the biscuits.

Regarding the results of antioxidant activity from the chemical extraction (Ext.2) (Figure 7b) initially, the different types of biscuits showed significant differences amongst each other. The biscuits prepared with FRPA (W, CA) had higher antioxidant activity than the control biscuits (B) and those formulated with reference fiber (P). During storage at room temperature, the values of antioxidant activity of all groups of biscuits decreased significantly. At the end of storage, the antioxidant activity values of the biscuits formulated with FRPA (W, CA) were still substantially higher than those of the control biscuits without fiber (B) and those formulated with pea fiber (P). 

When comparing the results obtained of Ext.1 (digestion) and Ext.2 (chemical extraction), it is confirmed as well that the digestion process liberates hydrolysable antioxidants, reactive with FRAP, from the plant structures, and therefore Ext.1 showed higher values.

The trends detected with the previously described methods were similar. The biscuits in accelerated storage had increased antioxidant activity, and the biscuits formulated with fiber (P, W, CA) had higher antioxidant activity than the control biscuits (B), due to the content of the fiber utilized. As was observed by Vitali et al. [30] the increase in antioxidant activity (in chemical and physiological extracts) is explained by the increase in extractable (or bioavailable) phenolic content during accelerated storage, due to the formation of certain products of the Maillard reaction.

**Figure 8:**
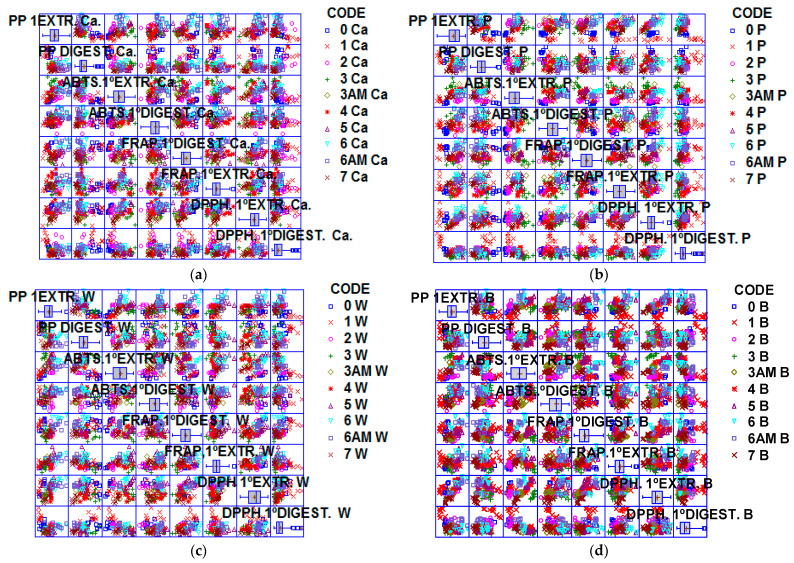
Matrix graphic of the biscuits studied (B, P, W, CA) during storage. (STATGRAPHICS Centurion^®^). (**a**) Biscuit CA (1% CaCl_2_∙5H_2_O), (**b**) Biscuit P (pea fiber), (**c**) Biscuit W (water), (**d**) Biscuit B (without fiber). Code codification: Letter (fiber source), number (months stored at room temperature), AM accelerated month).

The differences found between the values of antioxidant activity, obtained by the different antioxidant analysis methods, agreed with the studies performed by Rivero-Pérez et al. [63]. Finally, the increase in antioxidant activity could be correlated to a higher polyphenol content of FRPA of CA and W (Figure 8), having the biscuits formulated with FRPA-CA with the highest antioxidant activity. Finally, due to the moisture difference between biscuits it was noticed that the biscuits formulated with FRPA had a better water-holding capacity and kept the formulation water better during oven-baking. It may reduce heat on the surface of the biscuits, which may cause a loss in polyphenol content and antioxidant activity.

## 4. Discussion

The main sources of antioxidants in our diet are derived from plant-based foods and beverages. These dietary antioxidants are intricate combinations consisting of numerous compounds, and their cooperative effects are primarily manifested within the gastrointestinal tract. In addition to antioxidant vitamins (A, C, E) and carotenoids, plant-based foods encompass a wide variety of phenolic compounds that have the potential to boost the antioxidant capacity of our dietary intake [64,65]. Polyphenols, in fact, represent the predominant dietary antioxidants in terms of quantity and demonstrate a greater in vitro antioxidant capacity when compared to vitamins and carotenoids [66]. The collective synergistic effects of these dietary antioxidants are thought to contribute to the various health benefits associated with a well-balanced diet.

Polyphenols, as substantiated by research evidence [67], represent the prevailing antioxidants abundantly present in our dietary intake. Apart from their well-known antioxidant properties, polyphenols exhibit diverse specific biological activities that modulate gene expression [68], cellular signaling [69], and cell adhesion [70]. The scientific community demonstrates an escalating interest in investigating the biological properties of polyphenols concerning the prevention of age-related disorders, including cardiovascular disease and cancer [71]. To gain a comprehensive understanding of the pivotal role of polyphenols in human health, it is imperative to determine both the quantity of polyphenols ingested through the diet and their bioavailability.

Understanding the health effects of polyphenols poses a significant challenge due to the extensive presence of phenolic compounds in foods, each exhibiting distinct biological activities as demonstrated in various in vitro studies [49,72]. Furthermore, phenolic molecules are often unique to specific plant species, organs, or tissues, making it challenging to determine the precise composition of ingested polyphenols. Moreover, information regarding the overall consumption of polyphenols in diets is typically limited to specific compound categories like flavanols, flavones, catechins, or phenolic acids [49,73]. While numerous articles in the literature focus on specific types of polyphenols found in individual foods, there is a scarcity of information regarding the enrichment of staple products such as biscuits.

To study the content of polyphenols and its ability in our study, we employed the Folin-Ciocalteu method as our primary approach to quantify the total polyphenol content in our food matrix. It is important to note that most literature focusing on food polyphenols primarily concentrates on compounds that are dissolved in aqueous organic extracts, known as extractable polyphenols. However, this methodology has its limitations due to the extraction techniques employed. Certain polyphenols, particularly those with a high degree of polymerization or those associated with high molecular weight compounds, referred to as non-extractable polyphenols, may not be effectively captured using standard extraction methods. Nevertheless, this fraction of polyphenols found in our enriched food matrix may have the potential to exhibit bioactivity within the human gut once they are released from the food matrix through the action of digestive enzymes in the small intestine [49,74]. 

As mentioned before, to evaluate the bioavailability of the functional compounds of biscuits enriched with FRPA, a model of human digestion was carried out and an extract was obtained (Ext.1). In parallel, a chemical extraction was carried out of the same biscuits, to determine the bioavailable polyphenolic content, and two extracts were obtained for the determination of total polyphenol content: extractable (Ext.2) + hydrolysable (Ext.3).

Regarding polyphenols, significant differences were observed in the total polyphenol content between the biscuits before and after digestion (Ext.1), indicating the liberation of insoluble polyphenols bound to the plant structures during the acid extraction process. Similarly, biscuits formulated with fiber exhibited higher initial polyphenol content compared to control biscuits without fiber. This increase in polyphenol content can be attributed to the polyphenolic content provided by the added vegetable fiber in the formulation, as seen in Ext.1. Biscuits formulated with FRPA (W, CA) and pea fiber (P) had significantly higher initial polyphenol contents compared to biscuits formulated with P alone. 

During storage at room temperature, the concentration of extractable polyphenols (Ext.2 + Ext.3) significantly decreased by approximately 30% in all biscuit groups throughout the seven-month storage period. Biscuits enriched with FRPA (W, CA) exhibited higher total polyphenol content compared to the other biscuit types (B and P), showing a significant difference at the end of the storage period. Similar reductions in polyphenolic content were observed for all biscuit groups when stored at accelerated temperature. The accelerated temperature storage for three months resulted in a decrease in total polyphenol content, like the reduction observed after seven months of storage at room temperature for biscuit groups without FRPA (B and P).

All studied groups of biscuits correlated well with the content of total polyphenols, expressed as the sum of extractable soluble polyphenols (Ext.2) and the hydrolysable (Ext.3). However, the effect of acid hydrolysis, used for Ext.3, on the plant structures produced a greater liberation of polyphenolic compounds, leading to significant differences between the bioavailable and the total polyphenols. Ours results are supported by the data shown in (64) where polyphenols extracted from artichokes shown had good bioavailability after undergoing both in vitro digestion and Caco-2 human intestinal cell models (55.8% for total artichoke phenolics), proving that the polyphenol from artichoke could be bioavailable after digestion of biscuits formulated with FRPA specially the ones extracted with a solution of 1% (*w*/*w*) CaCl_2_∙5H_2_O. 

Consequently, both initially and during storage, the biscuits formulated with FRPA (W, CA) showed a higher content of bioavailable polyphenols compared to both the control biscuits without fiber (B) and the ones formulated with reference fiber (P, pea fiber), making the FRPA an excellent alternative to the use of reference fiber. 

The antioxidant activity results obtained from the different methods (ABTS.^+^, DPPH., and FRAP) show different values but the same trends in all the types of biscuits studied. They corresponded with the results achieved from the polyphenol content. Both initially and during storage, the biscuits formulated with FRPA (W, CA) showed higher antioxidant activity compared to both the control biscuits without fiber (B) and the biscuits formulated with reference fiber (P, pea fiber). In particular to DPPH., other authors used it to correlate the antioxidant capacity and polyphenol content when using functional plant-based ingredients [51] to enrich biscuits [53,54,55], showing similar overall antioxidant effects.

The antioxidant activity of the physiological extracts of digestion, obtained by the model of gastrointestinal digestion (Ext.1), was significantly higher than the activity found in the extracts obtained by chemical extraction (Ext.2 + Ext.3). Therefore, the use of chemical extraction methods only, for the estimation of antioxidant activity of cereal-based foods, underestimates the antioxidant activity of the substances liberated by the effect of gastrointestinal digestion. In conclusion, FRPA is an excellent source of bioavailable fiber with antioxidant activity, especially the FRPA (CA), and presents a good alternative to the use of reference fiber from peas. As per the findings of Saeed et al. [53], the antioxidant activity observed in the biscuit samples is attributed to the presence of antioxidants such us polyphenolic compounds and flavonoids. These antioxidants act as reducing agents, counteracting the formation of free radicals by donating electrons, which limits the production of peroxides and fat oxidation.

Apart from the known benefit for human health that the antioxidant effects of FRPA are not affected by the model of human digestion, proving its functional potential, using FRPA to enrich biscuits could also delay the oxidation process related to the rancidity of its fat composition.

Regarding moisture content, even when all the biscuits underwent similar process conditions (time-temperature), the biscuits formulated with artichoke fiber CA held significantly more moisture after the cooking process in the oven than the control biscuits. Similar results are also found in other publications [54], where enriched biscuit samples increased their water activity and moisture value. Increasing the water-holding capacity of the biscuits with FRPA could be interesting industrially because it could outperform commercial fibers with less water-holding capacity, like pea fiber, making cooking processes easier to control weight loss and excessive browning.

To determine their true potential as an alternative substitute for pea fiber in the bakery industry, we tested its sensorial acceptance. The sensory profile and textural stability of FRAP-enriched biscuits is already published in an article [75].

## 5. Conclusions

Both initially and during storage, the biscuits formulated with FRPA (W, CA) showed a higher content of bioavailable polyphenols and higher antioxidant activity compared to both control biscuits without fiber (B) and those formulated with commercial reference fiber (P, pea fiber). In conclusion, FRPAs might be a good source of fiber with antioxidant and bio-assimilable activity, especially FRPA (CA), which could be a valid alternative to commercial pea fiber in biscuit formulations.

## Figures and Tables

**Table 1:** Biscuit formulation.

Ingredient	%
Water	7.45
Glucose-fructose syrup	1.45
Sunflower oil	12.02
Whey	3.52
Sodium and ammonium bicarbonate	1
Salt	0.23
Lecithin	0.1
Wholemeal wheat flour	61.2
Fiber: Pea fiber (P) or FRPA (W, CA)	4
	100

## Data Availability

The data presented in this study are available on request from the corresponding author. The data are not publicly available due to privacy restrictions.
